# Probabilistic Risk Assessment of Heavy Metals in Mining Soils Based on Fractions: A Case Study in Southern Shaanxi, China

**DOI:** 10.3390/toxics11120997

**Published:** 2023-12-07

**Authors:** Lei Han, Yamin Fan, Rui Chen, Yunmeng Zhai, Zhao Liu, Yonghua Zhao, Risheng Li, Longfei Xia

**Affiliations:** 1 School of Land Engineering, Chang’an University, Xi’an 710054, China; 2021135016@chd.edu.cn (Y.F.); 2021135017@chd.edu.cn (Y.Z.); lz13@chd.edu.cn (Z.L.); yonghuaz@chd.edu.cn (Y.Z.); 2 Shaanxi Key Laboratory of Land Reclamation Engineering, Chang’an University, Xi’an 710054, China; 3 School of Earth Science and Resources, Chang’an University, Xi’an 710054, China; ruichen@chd.edu.cn; 4 Shaanxi Provincial Land Engineering Construction Group, Xi’an 710075, China; lirisheng1209@163.com (R.L.); summer321xia@foxmail.com (L.X.)

**Keywords:** heavy metals, fraction analysis, risk assessment, modified Hakanson index method, Monte Carlo simulation

## Abstract

With rapid economic development, soil heavy metal (HM) pollution has emerged as a global environmental concern. Because the toxicity of HMs differs dramatically among various fractions, risk assessments based on these fractions are of great significance for environmental management. This study employed a modified Hakanson index approach to evaluate the possible ecological impacts of soil HMs in a gold mine tailings pond in Shaanxi Province, China. A modified Hakanson–Monte Carlo model was built to perform a probabilistic risk assessment. The results showed that: (1) the exceedance rates of chromium (Cr) and zinc (Zn) were 68.75% and 93.75%, respectively. Moreover, the overall concentrations of nickel (Ni), copper (Cu), arsenic (As), and lead (Pb) were higher than the background soil environmental values in China. (2) HMs with the lowest oxidizable fraction were mostly present in the residual fraction. The oxidizable portions of Cr, Cu, and Pb and the reducible and residual fractions of As were notably distinct. (3) The risk degrees of Cr, Ni, Cu, and Zn were low; those of As and Pb were very high and moderate; and the comprehensive ecological hazard index was very high. This study offers a solid scientific foundation for ecological risk notification and environmental management.

## 1. Introduction

With rapid industrial development, soil heavy metal (HM) pollution caused by mining and the poor management of tailings has increasingly become a global environmental concern [1,2,3]. HMs are highly toxic, easily residual, and bioaccumulative [4,5], and their environmental impacts vary among the different HM fractions. In addition, the residual fraction maintains its chemical stability and biological activity by combining silicate minerals and crystalline ferromanganese oxides [6]. The reducible and oxidizable fractions are easily transformed into bioavailable fractions with environmental changes [7] that are hazardous to the environment. The acid-soluble fraction is the most bioavailable, as well as highly toxic [8] and highly migratory [9], and poses the greatest environmental risk. The fractions of HMs exhibit different behaviors in terms of migration enrichment, effectiveness, and potential toxicity [10,11], and ecological risk assessment is the basis and key to environmental management; therefore, risk assessments based on fractions are of great significance for ecological studies in mining areas [12].

In existing related studies, scholars have mainly used the risk assessment code (RAC) [13], phase and primary comparison [14,15], toxicity characteristic leaching procedure [16], and individual contamination factor [17] methods to assess the degree of ecological risk of HMs. Some studies have also improved existing methods, such as when Saeedi and Jamshidi-Zanjani [18] added the toxicity response factor of the Hakanson index method to the RAC method, and the modified RAC highlighted the aggregation and toxicity of HMs. Jiménez and Balderas [19] and Zhu et al. [20] modified the Hakanson index method, based on the relationship between exchangeable and carbonate-binding content and RAC risk classification. All the above methods are conventional deterministic risk assessment methods, but the selection of toxicity response coefficients and the randomness of monitoring data led to some uncertainty in the risk assessment results [21]. To address these problems, Monte Carlo simulations have been introduced into the risk assessments of soil and sediment HMs [21,22], organic matter [23,24], and new pollutants [25]. Probabilistic risk assessment has gradually become a new research trend [26]. However, the current probabilistic risk assessment is mainly conducted based on the total number of pollutants, without considering the influence of the fractions. Therefore, in this paper, we developed the modified Hakanson–Monte Carlo model to carry out a probabilistic risk assessment study based on the fractions of HM. The newly developed model not only considered the environmental risk and biotoxicity of different HM fractions, but also quantified the assessment uncertainty and accurately reflected the environmental risk status of HMs.

The Qinling Mountains represent a significant ecological security barrier for China and are a crucial global seed bank. They play an important role in the natural ecological environment on a global scale. However, in recent years, long-term mining resources and the accumulation of non-ferrous metal mine tailings ponds have damaged the ecological environment of the Qinling Mountains. Therefore, it is necessary to conduct a probabilistic risk assessment for HMs in the soil of the Qinling mining area. Additionally, the principal aims of this research are as follows: (1) to measure the concentration and investigate the distribution of chromium (Cr), zinc (Zn), nickel (Ni), copper (Cu), arsenic (As), and lead (Pb) in the gold mine tailings pond; (2) to measure the concentration and investigate the distribution of the fractions (acid/water-soluble, reducible, oxidizable, and residual fractions); (3) assess the characteristics of HM contamination and potential risk using the Hakanson index and the modified Hakanson index methods; and (4) to construct the modified Hakanson–Monte Carlo model and conduct the probabilistic risk assessment of HMs in mining soils, which is of guiding significance for environmental management and precise risk control. This study provides the scientific basis for assessing the characteristics of HM pollution, carrying out environmental management, and precise risk control.

## 2. Materials and Methods

### 2.1. Study Area

The study area is located in the southern part of the Qinling Mountains, in the southeast of Shaanxi Province, and in the southwest of Shangluo City. It is between 33°2′30″ and 34°24′40″ N and 108°34′35″ and 109°36′51″ E and the climate is subtropical semi-humid. The annual average sunshine is 1947.4 h, the annual average temperature is 12.2 °C, and the annual average precipitation is 804.8 mm. The gold mine in the study area was put into operation in 1993 and the “4.30” dam collapse accident occurred in 2006. Both the tailings spill and abandoned slag left behind have caused some degree of HM contamination in the surrounding farmland. The soil type is yellow-brown loam, with a thin layer of poorly cultivated soil, low organic matter content, low fast-acting nutrient content, and an imbalance in the nitrogen and phosphorus ratio [27].

### 2.2. Sample Collection and Pretreatment

**Figure 1:**
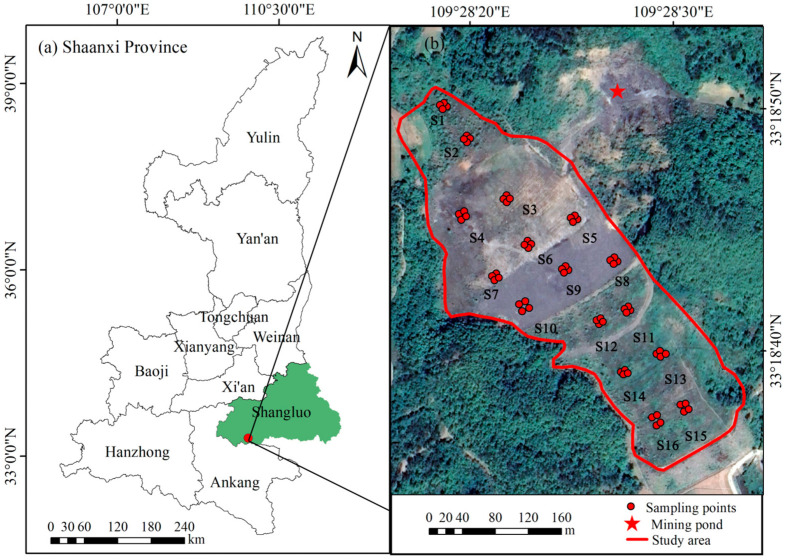
Location of the study area: (**a**) location of Shangluo City in Shaanxi Province; (**b**) distribution of sampling points.

In September 2020, 16 sampling points (S1–S16) were established (Figure 1), using the grid placement method. One kilogram of topsoil (0–20 cm) was collected from each point along the diagonal of the grid, mixed in equal amounts, and placed in a self-sealed bag. After removing stones, plant residues, and organic debris from the soil samples, approximately 300 g of each soil sample was retained, according to the quadratic method. The soil samples were air-dried at a room temperature of 25 °C, milled, and screened through a 100-mesh nylon sieve (sieve opening: 0.149 mm). All soil samples were placed in labeled polyethylene bags and then taken back to the laboratory for the determination of soil chemical properties and HM content.

### 2.3. Chemical Analysis and Instrument Measurement

#### 2.3.1. Determination of HM Content in Soil

Soil samples (0.1000 g) were accurately weighed and separately digested with 1 mL HCl + 4 mL HNO_3_ + 1 mL HF + 1 mL H_2_O_2_ in Teflon digestion tubes. The concentration was determined using an atomic fluorescence spectrometer (AFS-9800, Haiguang, Beijing, China) [28]. The concentrations of Cr, Ni, Cu, Zn, and Pb were determined using inductively coupled plasma mass spectrometry (ICP-MS, Agilent 7500c, Santa Clara, CA, USA) [29].

#### 2.3.2. Determination of the Fractions of HMs in Soil

The morphology of HMs primarily refers to their valence states, tautomers, and fractions. HM fractions are chemical morphologies formed by the reaction of metal ions in the environment with the soil via adsorption and complexation, which are important parameters for assessing environmental effects [30]. The modified BCR method [31,32,33] was used to continuously extract HM fractions, including acid/water-soluble (F1), reducible (F2), oxidizable (F3), and residual (F4) fractions. The detailed extraction procedures are listed in Table S1. Different fractions of the extracts produced during the sequential extractions were analyzed using ICP-MS (Agilent 7500c, Santa Clara, CA, USA).

#### 2.3.3. Quality Control and Assurance

Standard reference materials (GBW07401–GBW07408, Chinese standardized reference materials for soils [34]) were regularly used for strict quality control throughout the analysis. Additionally, the recoveries of HMs and fractions were 82.43–109.14% and 95.85–105.06%, respectively.

### 2.4. Pollution Assessment of HMs

#### The Geoaccumulation Index

The geoaccumulation index method was first proposed by Müller [35] for the characterization of HM contamination in sediments and is now widely used for soil HM contamination and ecological risk assessment [36,37]. While considering the influence of sedimentary diagenesis on background values, the method also fully reflects the influence of HM contamination from human activities and is an important parameter for distinguishing the effects of human activities. The calculation formula is as follows:

(1) $$I_{geo}={log}_{2}\left(\frac{C_{n}}{1.5B_{n}}\right)$$

where $I_{geo}$ is the geoaccumulation index; $C_{n}$ is the measured concentration of HMs in the soil (mg/kg); and $B_{n}$ is the reference value of HMs (mg/kg). In this study, we took the environmental background value of Shaanxi Province as the reference value, and the background values of Cr, Ni, Cu, Zn, As, and Pb were 67.0, 33.9, 26.4, 72.2, 14.10, and 17.0 mg/kg, respectively [38]. The geoaccumulation index values and the classification of the HM pollution degree are shown in Table 1 [39].

### 2.5. Potential Ecological Risk Assessment

#### 2.5.1. The Modified Hakanson Index

(1) Concentration correction based on HM fractions

The potential ecological hazard index method was established by Hakanson and based on sedimentological principles to comprehensively evaluate the quality of HM pollution in water sediments [25]. This method was later used in studies related to soil HM pollution [40]. This can comprehensively reflect the potential environmental impacts of HMs [41]. Some scholars have improved the Hakanson index method by introducing fractions. For example, Jiménez and Balderas [19] and Zhu et al. [20] corrected the concentrations using toxicity coefficients based on the contents of exchangeable and carbonate-bound fractions. Li et al. [42] used the sum of the exchangeable and bound carbonate fractions as the corrected concentration. Wang [7] assigned the corresponding factors to the fractions and summed them according to the difference in biological toxicity risk.

According to the different impacts of the fractions on the environment, combined with the above-mentioned studies, the toxicity coefficient θ was used to characterize the contribution of the assigned fraction to the ecological risk, and the values of $\theta_{1}$, $\theta_{2}$, and $\theta_{3}$ were 1.6, 1.0, and 0.6 [7,20,42,43], respectively. In addition, the corrected concentration ($C_{r}^{i})$ at individual sample sites was lower than the background value and did not meet the conditions of the Hakanson index method. Therefore, the secondary correction of $C_{r}^{i}$ was calculated using Equation (2) [43]. The calculation formulas for the single and comprehensive potential ecological hazard indices of HMs are as follows:

(2) $$C_{r}^{i}=\theta_{1}C_{r1}^{i}+\theta_{2}(C_{r2}^{i}{+C}_{r3}^{i})+\theta_{3}C_{r4}^{i}$$

(3) $$C_{r}^{i\prime }={|C}_{r}^{i}-C_{0}^{i}|+C_{0}^{i}$$

where $C_{r1}^{i},C_{r2}^{i},C_{r3}^{i},andC_{r4}^{i}$ are the acid-/water-soluble, reducible, oxidizable, and residual fractions, respectively (mg/kg). ${C_{r}^{i},C}_{r}^{i\prime }$ is the content of the corrected concentration of the fraction (mg/kg). $C_{0}^{i}$ is the reference value. The environmental background values of Cr, Ni, Cu, Zn, As, and Pb in Shaanxi Province were 67.0, 33.9, 26.4, 72.2, 14.10, and 17.0 mg/kg, respectively [38].

(4) $$C_{f}^{i}=C_{r}^{i\prime }/C_{0}^{i}$$

(5) $$E_{r}^{i}=T_{r}^{i}\times C_{f}^{i}$$

(6) $$RI=\sum_{i=1}^{n}E_{r}^{i}$$

where $C_{f}^{i}$ is the HMs pollution factor, $T_{r}^{i}$ is the toxicity response value of HMs, and $T_{r}^{i}$ was determined to be Cr = 2, Ni = Cu = 5, Zn = 1, As = 10, and Pb = 5 [41,44]. $E_{r}^{i}$ and RI denote the single and comprehensive potential ecological hazard indices of HM, respectively.

(2) Grading criteria correction for $E_{r}^{i}$ and RI

Hakanson [45] assessed the potential ecological risk of contaminants in sediments. The lowest degree of the upper limit of $E_{r}^{i}$ was the maximum toxicity response factor of the contaminant, assuming there was no contamination of the contaminant ($C_{f}^{i}$ = 1). Of the six HMs involved in this study, As had the largest toxicity response coefficient; therefore, the lowest upper limit of $E_{r}^{i}$ was 10. RI grading criteria are related to the type and quantity of HMs [38]. According to Ma et al. [46] and Li et al. [47], the lowest limit value of RI was divided by the total value of the toxicity coefficients of the eight pollutants, using the Hakanson index method, to obtain the unit toxicity coefficient. The sum of the toxicity response coefficients of the six HMs was 28, and the two were multiplied and taken as 10 integers to 30; thus, the lowest RI upper limit was 30. The modified Hakanson index grading criteria are presented in Table S2.

#### 2.5.2. Monte Carlo Simulation

A Monte Carlo simulation is based on the statistical knowledge of mathematics and probability and is a commonly used method for probabilistic risk assessment [48]. In this study, we defined random variables based on $C_{r}^{i\prime }$ distribution and the predicted values in terms of $C_{r}^{i\prime },E_{r}^{i},$ and RI relationships. We also established a probabilistic simulation between the variables and the predicted values by setting the simulation parameters (50,000 samplings, 95% confidence interval). Random sampling was then used to obtain $E_{r}^{i}$ and RI prediction values, which were used for risk assessment to improve the accuracy of risk assessment [49].

### 2.6. Parameter Selection

HM contamination of soil is generally judged by comparing the HM contents with the environmental background values of the soil; therefore, the selection of suitable parameters is the key to scientifically assessing HM pollution characteristics. In this study, soil environmental background values [50] were selected as the parameters, and the background values of Cr, Ni, Cu, Zn, As, and Pb are listed in Table S3.

## 3. Results and Analysis

### 3.1. Characteristics of HMs

**Figure 2:**
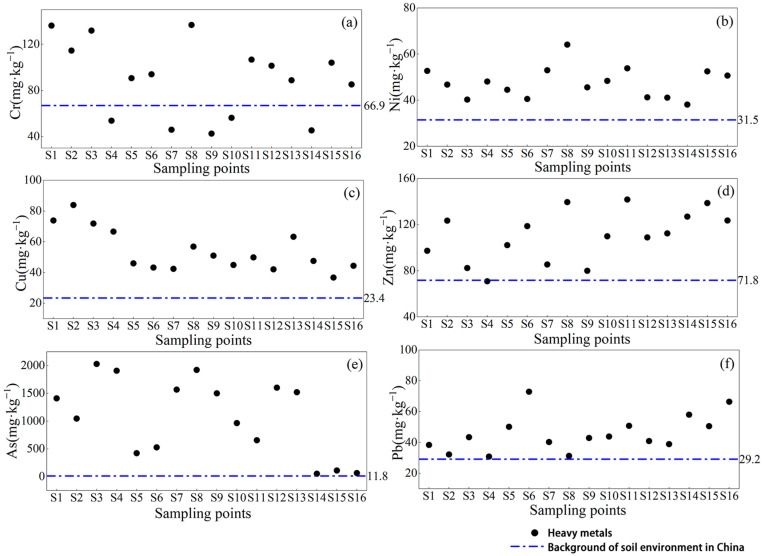
Distribution of HMs in the study area: (**a**) Cr concentration; (**b**) Ni concentration; (**c**) Cu concentration; (**d**) Zn concentration; (**e**) As concentration; and (**f**) Pb concentration.

The results of the soil HM distribution at the sampling sites in the study area are shown in Figure 2. The exceedance rate refers to the ratio of the number of sampling points where the total amount exceeds the environment background value in China to the total number of sampling points. It is generally believed that the higher the exceedance rate of HMs, the higher the HM contamination in the study area. The exceedance rate of Cr was 68.75%, the concentration of Zn was lower than the soil background values of China only at S4, and the concentrations of Ni, Cu, As, and Pb at the sampling sites exceeded the background values for the soil environment in China. The mean Cr, Ni, Cu, Zn, As, and Pb contents were 1.34, 1.51, 2.31, 1.53, 91.60, and 1.57 times, respectively. The coefficient of variation is a statistical parameter that characterizes the ratio of the standard deviation to the mean concentration, reflecting the uniformity and variability of the HM distribution. The larger the coefficient of variation, the more significant the dispersion of the spatial distribution of HMs [51]. As shown in Table S4, the coefficients of variation (CV) of the concentrations of HMs were as follows: As > Cr > Cu = Pb > Zn > Ni. The coefficient of variation of As was higher than those of the other metals and the dispersion of the spatial distribution was significant. In addition, the coefficient of variation for Ni was the smallest, and the spatial distribution was homogeneous.

### 3.2. Characteristics of HM Fractions

#### 3.2.1. Distribution of HM Fractions

As shown in Table S5, the distributions of different HM fractions differed. The CV of the acid/water-soluble content of Ni was the highest at 79.42%. The CVs of the reducible and residual As were 109.51% and 108.90%, respectively, which indicated strong variability. The fractions of Cr, Cu, Zn, and Pb had the largest CVs for the oxidizable content, and only Zn was moderately variable. Except for Zn, the CVs of the oxidizable contents of Cr, Cu, and Pb exceeded 100%, indicating strong variability with significant differences in spatial distribution.

The distribution of the HM fractions was obtained using a Shapiro–Wilk test, and only the reducible fraction of As showed an abnormal distribution. According to statistical knowledge and the distribution of the boxplot, the reducible fraction of As at S4 was anomalous. After the outliers were removed, all fractions obeyed a normal or lognormal distribution, which satisfied the characteristics of a normal distribution, and the mean and standard deviation could be used to explain the whole distribution.

#### 3.2.2. Content of HM Fractions

**Figure 3:**
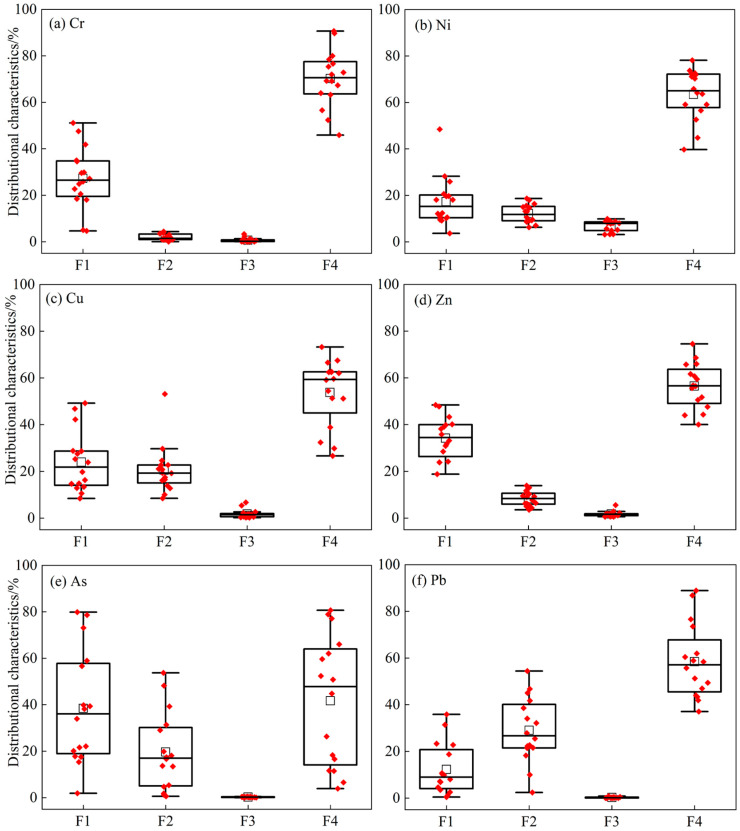
Boxplots of the fraction concentrations for HMs in the study area (F1: acid/water-soluble fraction; F2: reducible fraction; F3: oxidizable fraction; F4: residual fraction. The red and box represent data points and the mean value, respectively).

The proportions of Cr, Ni, Cu, Zn, As, and Pb are shown in Figure 3. Based on the mean HM contents, the distributions of Cr, Ni, Cu, Zn, and As were generally similar in the study area, and the contents of all fractions were as follows: F4 > F1 > F2 > F3. Additionally, the contents of Cr, Ni, Cu, Zn, and As in the residual fraction were 45.91–90.66%, 39.70–78.15%, 26.63–73.23%, 40.02–74.56%, and 3.87–80.65%, respectively. Except in the residue, the acid/water-soluble fractions of Cr, Ni, Cu, Zn, and As were high. The mean contents of Cr, Ni, Cu, Zn, and As in the acid/water-soluble fractions were 27.29%, 17.31%, 23.95%, 34.20%, and 38.43%, respectively. The fraction content of Pb decreased in the order of F4 > F2 > F1 > F3, and the content of Pb in the residual fraction ranged from 37.06% to 88.88%. Reducible Pb was the most abundant fraction, except for residual Pb. Comparatively, the acid/water-soluble fractions of Cr, Ni, Cu, Zn, and As were higher than those of Pb, while the proportion of Pb in the reducible fraction was higher than that of the remaining five metals.

### 3.3. Pollution Assessment of HMs

The mean values of the geoaccumulation index and pollution degree are shown in Table 2. The mean values of the six HMs were in the following order: As > Pb > Cu > Zn > Ni > Cr. Among them, As had the highest geoaccumulation index, and the pollution degree was extremely polluted. It was followed by Pb, Cu, and Zn, with a lightly polluted degree of contamination. The geoaccumulation indices of Ni and Cr were less than 0, and the pollution degree was unpolluted.

The pollution degree percentages of the geoaccumulation index in the study area are shown in Table 3. The pollution degree of As was in the extremely polluted, heavily to extremely polluted, moderately polluted and moderately to heavily polluted ranges, with percentages of 62.5%, 18.75%, 12.5% and 6.25%, respectively. Metal Pb was in the lightly polluted and moderately polluted degrees of 81.25% and 18.75%, respectively, while Cu was in the unpolluted, lightly polluted, and moderately polluted degrees of 6.25%, 87.5%, and 6.25%, respectively. The pollution degrees of Cr, Ni, and Zn were in the unpolluted and lightly polluted, and the lightly polluted percentages were 43.75%, 31.25%, and 62.5%, respectively.

### 3.4. Potential Ecological Risk Assessment

#### 3.4.1. Hakanson Index Method

**Figure 4:**
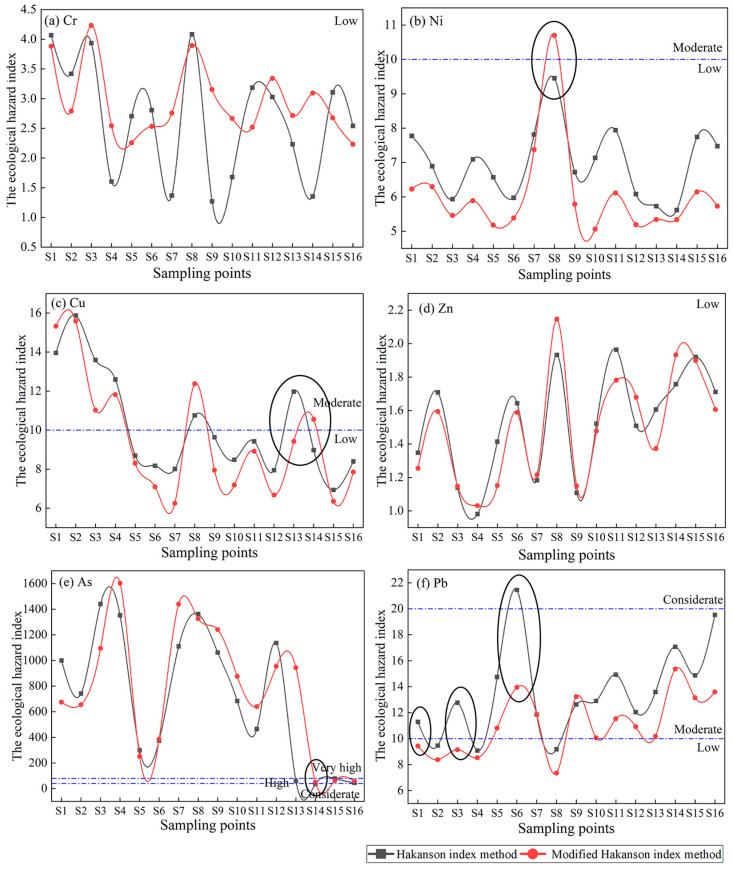
Ecological hazard index of HMs using two index methods. (Data in black circles indicate that this sampling point had different risk degrees based on the two index methods).

In this study, the ecological hazard indices of the six HMs were calculated based on the Hakanson and modified Hakanson index methods. Furthermore, ecological risk classification was based on the relationship between the value of the ecological hazard index and the modified classification criteria (Figure 4). The mean values of the ecological hazard index were as follows: As > Pb > Cu > Ni > Cr > Zn, with risk degrees of very high, moderate, moderate, low, low, and low, in that order. The mean value of the comprehensive ecological hazard index (RI) was 801.43, and the risk degree was very high.

As shown in Table S6, the ecological hazard indices of As ranged from 36.25 to 1440.21, with risk degrees including very high, high, and considerable, accounting for 81.25%, 12.5%, and 6.25%, respectively. The ecological hazard indices of Pb ranged from 9.08 to 21.45, with low, moderate, and considerable values accounting for 18.75%, 75%, and 6.25%, respectively. The ecological hazard indices of Cu ranged from 6.94 to 15.87, with 62.5% and 37.5% for low and moderate hazard, respectively. The ecological hazard indices of Cr, Ni, and Zn were 1.27–4.08, 5.62–9.45, and 0.98–1.96, respectively. The degrees of RI risk were very high and high, accounting for 81.25% and 18.75%, respectively.

#### 3.4.2. Modified Hakanson Index Method

The mean values of the ecological hazard index followed the descending order of As > Pb > Cu > Ni > Cr > Zn, with risk degrees of very high, moderate, low, low, low, and low, in that order. The mean RI was 797.00, and the risk degree was very high.

The ecological hazard indices of As ranged from 49.87 to 1602.96, with the risk degrees of very high and high accounting for 81.25% and 18.75%, respectively. The ecological hazard indices of Ni ranged from 5.06 to 10.70, with low and moderate percentages of 93.75% and 6.25%, respectively, and the ecological hazard indices of Cr and Zn were 2.23–4.23 and 1.03–2.15, respectively, with a low risk. The percentages of very high and high comprehensive ecological hazard indices were 81.25% and 18.75%, respectively.

#### 3.4.3. Probabilistic Risk Assessment

Combined with the ecological risk classification criteria in Table S2, the probabilities of the six HM ecological hazard indices at different degrees of risk and the cumulative probability distributions of the ecological hazard indices are shown in Table S7 and Figure S1, respectively.

According to Table S7, the probability of Cr and Zn being low was 100%. Therefore, the risk degree of Cr and Zn was low, and the distribution of risk levels for the two HMs was relatively concentrated. The ecological hazard index of Pb was low and moderate, with probabilities of 36.15% and 63.85%, respectively; thus, the risk degree of Pb was moderate. Both Ni and Cu had low, moderate, and considerable spatial distributions. According to the probability magnitude, it was known that the risk degree of both Ni and Cu was low. For As and RI, the probability of being very high was the largest of the five classes involved; therefore, both As and RI were very high and the spatial distribution was more significant.

The ecological hazard index is an important indicator for ecological risk assessment, and its value directly determines the degree to which a region is located [21]. To investigate the differences in the contribution of different HMs to the ecological hazard index, a sensitivity analysis of the comprehensive ecological hazard index was conducted using Crystal Ball 11. As shown in Figure S2, the contributions of different HMs followed the descending order As > Pb > Cu > Ni > Cr > Zn.

## 4. Discussion and Conclusions

### 4.1. Discussion

#### 4.1.1. Characteristics of HMs

The maximum exceedance times of Cr, Ni, Cu, Zn, and Pb were 2.04, 2.03, 3.59, 1.97, and 2.50 times, respectively, indicating that the soil was contaminated with Cr, Ni, Cu, Zn, and Pb to some extent in the study area. The maximum exceedance multiple of As ranged from 4.33 to 172.09, indicating that metal As was the main element that might be a risk to the soil ecology in the study area. Chen et al. [27] showed that the main components of pollution in the mining area were As, Cd, Cr, and Hg. However, the degree of Cr differed from that of this study, probably because of the lower content of Cr in the non-residue fraction and the lower risk assessment rating based on the fractions. The variability in soil properties is related to the physical and chemical properties of the soil and human activities, and the coefficient of variation varies with human activities [51]. As shown in Table S4, the coefficients of variation for the concentrations of HMs ranged from 14% to 64%. The coefficient of variation values of As were higher than those of the other metals, and the total amount showed a basic decreasing trend as the distance from the tailings pond increased, especially for S14–S16. This indicates that the spatial distribution of metallic As in the study area was significantly discrete and that metallic As was highly susceptible to human activity factors, such as mining [52,53].

#### 4.1.2. Characteristics of HM Fractions

HMs in soil exist in an ionic state in solution, and the stable state is adsorbed onto the surface of soil minerals, organic matter, and their complexes [54]. Comparing the mean contents of the fractions in the study area, HMs were mainly present in the residual fraction, and the results of this study are consistent with those of previous studies [55,56]. This indicates that metals are strongly associated with the mineral crystal structure and are present in the environment in a stable fraction with minimal environmental risk [57,58]. The oxidizable proportions of the six HMs were lower than their reducible proportions. According to Liu et al. [59], if the soil organic matter content is low, the organic matter cannot compete effectively with Fe–Mn oxides. The content of organic matter in the study area ranged from 0.03 to 5.28 g/kg, and it was at the sixth level of the National Soil Nutrient Census, which resulted in a high proportion of reducible fraction in the soil. Moreover, sample sites S3–S12 belong to the zone of intense human activities, and most of them had high acid/water-soluble contents of As, which may pose a high potential risk to the ecological environment. In addition to the above characterization, we also found the CVs of the reducible and residual As were all over 100%, which was strong variability. According to Wang et al. [51], soil parent material, irrigation, and human activities are the main causes of soil property variability. It is generally also accepted that the higher the coefficient of variation, the greater the influence of human activities. Therefore, we considered that the contents of the reducible and residual As were mainly influenced by local mining activities.

#### 4.1.3. The Reasonability of the Modified Hakanson Index Method

In this study, the Hakanson index and the modified Hakanson index methods were used to assess the potential ecological risk of HMs in the soil of mining areas. The assessment results of the two index methods were consistent, but there were also some differences. For Ni at S8 and Cu and As at S14, the risk degree of the modified Hakanson index method was higher than that of the Hakanson index method, whereas the risk level of the Hakanson index method was high for Cu at S13 and Pb at S1, S3, and S6. As shown in Figure 2, the total concentrations of the corresponding metals at the sampling sites exceeded the background values for the soil environment in China. However, the former were higher in the acid/water-soluble fraction (48.48%, 49.21%, and 78.61%) and had a high potential for migration into the environment, whereas the latter were lower in the non-residual fraction and had a lower impact on the environment. The environmental risk of soil HMs is not only related to the total concentration of HMs, but also to the content of the fractions. If only the total HM concentration is considered, the degree of risk may be too high [60,61]. Therefore, the modified Hakanson index method is more accurate for these sample sites. Compared to the Hakanson index method, the modified Hakanson index method considers the effects of both HM toxicity and deposition patterns. Therefore, the modified Hakanson index method was highly suitable for risk assessment in the study area.

#### 4.1.4. Correlation Analysis between the Fractions of As and Soil Physicochemical Properties

In this study, we determined the content of soil physicochemical properties such as soil organic matter, pH, total nitrogen, total phosphorus, total potassium, alkali-hydrolyzable nitrogen, available phosphorus, and rapidly available potassium. The organic matter content in the study area ranged from 0.03 to 5.28 g/kg with a mean value of 1.56; the pH ranged from 7.67 to 8.89 with a mean value of 8.43, which is weakly alkaline or alkaline. According to the National Second Soil Nutrient Classification Standard, the organic matter content belonged to the sixth standard level and the soil fertility was low. The mean content of total nitrogen and alkali-hydrolyzable nitrogen belonged to the third and fourth standard levels, but the contents varied greatly among different sampling points. The total and available phosphorus contents of all sampling sites belonged to the sixth standard level, and the soil nutrients were low. The total potassium content belonged to the sixth standard level, and the mean content of rapidly available potassium was 78.28 mg/kg, which belonged to the fourth standard level, while the maximum and minimum contents belonged to the first and sixth standard levels, respectively.

**Figure 5:**
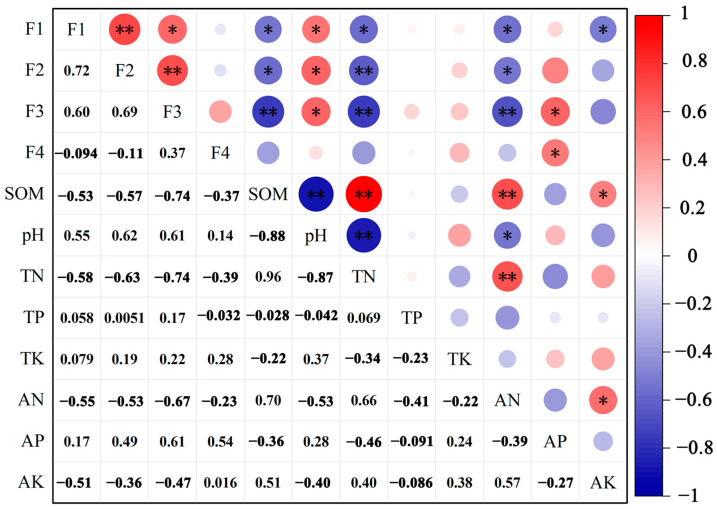
Correlation analysis between the fractions of As and soil physicochemical properties. (* and ** represent two different significance levels of *p* ≤ 0.05 and *p* ≤ 0.01, respectively; SOM: soil organic matter; TN: total nitrogen; TP: total phosphorus; TK: total potassium; AN: alkali-hydrolyzable nitrogen; AP: available phosphorus; AK: rapidly available potassium).

The correlation analysis between the fractions of As and soil physicochemical properties is shown in Figure 5. According to the correlation analysis, the acid/water-soluble fraction of As showed a significant negative correlation (*p* ≤ 0.05) with organic matter, total nitrogen, alkali-hydrolyzable nitrogen, and rapidly available potassium, and a significant positive correlation (*p* ≤ 0.05) with pH. The content of As in the reducible fraction showed a significant negative correlation with organic matter and alkali-hydrolyzable nitrogen (*p* ≤ 0.05), a significant positive correlation with pH (*p* ≤ 0.05), and a highly significant negative correlation with total nitrogen (*p* ≤ 0.01). The oxidizable fraction of As showed highly significant negative correlations with organic matter, total nitrogen, and alkali-hydrolyzable nitrogen (*p* ≤ 0.01), and significant positive correlations with pH and available phosphorus (*p* ≤ 0.05). The content of As in the residual fraction only showed a significant positive correlation with available phosphorus (*p* ≤ 0.05), and the correlation with other soil physicochemical properties was weak and had not reached a significant level (*p* > 0.05). Liu et al. [59] pointed out that, once human activities change the redox conditions of the soil environment, the reducible and oxidizable fractions are released into the environment, posing a serious threat to the soil and the ecological environment. Li et al. [62] found that, when the organic matter content increased, part of the oxidizable Pb was converted to the reducible fraction. In conclusion, changes in soil environmental conditions can affect the distribution of HM fractions and interconversion between fractions [63,64,65].

#### 4.1.5. The Probabilistic Risk Assessment of HMs

HMs have a certain accumulative nature when the HM content in the soil around a mine exceeds the environmental background values [66,67]. The accumulation of excessive HMs can directly affect the environment through the migration and transformation of soil–plant systems [68,69]. The results of the geoaccumulation index calculations showed that the pollution degree of As was extremely polluted, Cu, Zn and Pb were lightly polluted and Cr and Ni were unpolluted. However, the degrees of risk for Cr, Ni, Cu, Zn, and Pb were low or moderate in Table S7. There are differences in the assessment results of the two methods, which may be due to differences in raw data and assessment grading, but since the two methods have their own characteristics, the assessment results can complement each other and reflect the degree of contamination and the risk situation more realistically. The high ecological risk of As is influenced by the mining process on the one hand [70], and the accumulation of tailings aggravates the degree of soil contamination. On the other hand, it was related to the large toxicity response coefficient of As. In addition, by exploring the differences in the contributions of different HMs to the RI (Figure S2), the contribution of As was 98.06%, indicating that As was the main pollutant in the study area. Considering the more severe soil As pollution in the study area, the acid/water-soluble content of As was also higher. Tu et al. [6] pointed out that the acid-soluble fraction is more mobile and directly bioavailable. Wang [7] also pointed out that the exchangeable fraction is sensitive to environmental changes, is easy to migrate and transform, and can be absorbed by plants. The correlation analysis between the fractions of As and the soil physicochemical properties also showed a significant correlation between the acid/water-soluble and soil organic matter and pH. Therefore, the exchange content of HMs in the soil can be reduced by adjusting the pH and increasing the organic matter content, thus reducing the environmental risk of HMs and providing a scientific basis for mine ecological environment management and soil pollution prevention. In addition, in our future research work, we will prepare appropriate sampling programs according to the local conditions of the study area, and we will also communicate smoothly with the residents in advance, in order to make our best efforts to ensure the smooth implementation and completion of the sampling and research work.

### 4.2. Conclusions

(1) The exceedance rate of Cr was 68.75%, that of Zn was more than 90%, and the total Ni, Cu, As, and Pb contents exceeded the soil environmental background value. Metal As had the largest exceedance rate and coefficient of variation, indicating that the content of As was greatly influenced by human activities and was the main element that may be at risk in the soil ecosystem of the study area.

(2) HMs exist mainly in the stable residual fraction, with the lowest amount in the oxidizable fraction. Due to the low organic matter content in the study area, the oxidizable fraction contents of HMs are all lower than the reducible fraction. In addition, changes in soil physical and chemical properties also affect the distribution and transformation of fractions.

(3) The results of HM contamination showed that the pollution degree of As was extremely polluted, Cu, Zn, and Pb were lightly polluted, and Cr and Ni were unpolluted. The more serious degree of As in the study area suggests that local soil environmental quality testing should be enhanced to varying degrees depending on the proximity to the tailings pond.

(4) The risk degrees of Cr, Ni, Cu, and Zn were low, whereas those of Pb were moderate. Cr and Zn had concentrated spatial distributions, whereas Ni and Cu had specific spatial distribution characteristics. The degrees of As and RI were very high, with significant dispersion in spatial distribution. As the main pollution factor in the study area, relevant departments can implement mine ecological environment management by implementing targeted measures.

## Figures and Tables

**Table 1:** The geoaccumulation index and the classification of pollution degree.

I_geo_	≤0	0–1	1–2	2–3	3–4	4–5	≥5
Pollution Degree	Unpolluted	Lightly polluted	Moderately polluted	Moderately to heavily polluted	Heavily polluted	Heavily to extremely polluted	Extremely polluted

**Table 2:** The mean value of the geoaccumulation index and pollution degree.

HMs	Cr	Ni	Cu	Zn	As	Pb
I_geo_	−0.17	−0.10	0.45	0.02	5.68	0.84
Pollution Degree	Unpolluted	Unpolluted	Lightly polluted	Lightly polluted	Extremely polluted	Lightly polluted

**Table 3:** The pollution degree percentage of the geoaccumulation index (%).

Pollution Degree	Cr	Ni	Cu	Zn	As	Pb
Unpolluted	56.25	68.75	6.25	37.5	0	0
Lightly polluted	43.75	31.25	87.5	62.5	0	81.25
Moderately polluted	0	0	6.25	0	12.5	18.75
Moderately to heavily polluted	0	0	0	0	6.25	0
Heavily polluted	0	0	0	0	0	0
Heavily to extremely polluted	0	0	0	0	18.75	0
Extremely polluted	0	0	0	0	62.5	0

## Data Availability

Data are contained within the article and Supplementary Materials.

## Supplementary Materials

The following supporting information can be downloaded at: https://www.mdpi.com/article/10.3390/toxics11120997/s1, Figure S1: Cumulative frequencies distribution of ecological risk index of (a) Cr, Ni, Cu, Zn and Pb; (b) As and RI; Figure S2: Sensitivity analysis of comprehensive ecological hazard index; Table S1. Modified BCR sequential extraction procedure [31,32,33]; Table S2: The modified Hakanson index method risk degree classification standard [46,47]; Table S3: Environmental background values for heavy metals (HMs) in yellow-brown loam [50]; Table S4: Concentrations of total heavy metals in the study area; Table S5: Concentrations of the heavy metal fractions in the study area; Table S6: Comparison of potential ecological risk of HMs in soil based on the Hakanson index and modified Hakanson index; Table S7: Probability of the ecological hazard index of HMs at different risk degrees (%).

## References

[1] Lian M., Sun L., Sun T., Tang J. (2013). New Research Development on Heavy Metals’ Speciation in Soil. Appl. Mech. Mater..

[2] Li H., Yao J., Min N., Chen Z., Li M., Pang W., Liu B., Cao Y., Men D., Duran R. (2022). Comprehensive evaluation of metal(loid)s pollution risk and microbial activity characteristics in non-ferrous metal smelting contaminated site. J. Clean. Prod..

[3] Luo X., Wu C., Lin Y., Li W., Deng M., Tan J., Xue S. (2023). Soil heavy metal pollution from Pb/Zn smelting regions in China and the remediation potential of biomineralization. J. Environ. Sci..

[4] Jiang X., Zou B., Feng H., Tang J., Tu Y., Zhao X. (2019). Spatial distribution mapping of Hg contamination in subclass agricultural soils using GIS enhanced multiple linear regression. J. Geochem. Explor..

[5] Shen F., Mao L., Sun R., Du J., Tan Z., Ding M. (2019). Contamination Evaluation and Source Identification of Heavy Metals in the Sediments from the Lishui River Watershed, Southern China. Int. J. Environ. Res. Public Health.

[6] Tu J., Zhao Q., Yang Q. (2012). Morphological distribution of heavy metals in sludge from urban wastewater treatment plants and its potential ecological risk assessment in Northeast China. Acta Sci. Circumst..

[7] Wang X. (2012). Simulation of ecological hazard index based on a heavy metal polymorphic-biotoxic activity weighting system. J. Henan Norm. Univ. (Nat. Sci. Ed.).

[8] Singh K.P., Mohan D., Singh V.K., Malik A. (2005). Studies on distribution and fractionation of heavy metals in Gomti river sediments—A tributary of the Ganges, India. J. Hydrol..

[9] Mcgrath S.P., Cegrarra J. (1992). Chemical extractability of heavy metals during and after long-term applications of sewage sludge to soil. J. Soil Sci..

[10] Shaheen S.M., Rinklebe J. (2014). Geochemical fractions of chromium, copper, and zinc and their vertical distribution in flood-plain soil profiles along the Central Elbe River, Germany. Geoderma.

[11] Sundaray S.K., Nayak B.B., Lin S., Bhatta D. (2011). Geochemical speciation and risk assessment of heavy metals in the river estuarine sediments—A case study: Mahanadi basin, India. J. Hazard. Mater..

[12] Hu B., Guo P., Su H., Deng J., Zheng M., Wang J., Wu Y., Jin Y. (2021). Fraction distribution and bioavailability of soil heavy metals under different planting patterns in mangrove restoration wetlands in Jinjiang, Fujian, China. Ecol. Eng..

[13] Pejman A., Bidhendi G., Ardestani M., Saeedi M., Baghvand A. (2017). Fractionation of heavy metals in sediments and assessment of their availability risk: A case study in the northwestern of Persian Gulf. Mar. Pollut. Bull..

[14] Sun J., Yu R., Hu G., Su G., Wang X. (2017). Assessment of heavy metal pollution and tracing sources by Pb & Sr Isotope in the soil profile of woodland in Quanzhou. Environ. Sci..

[15] Chen J., Liu B., Cai L., Wang G., Yin K., Chen H., Li Z. (2018). Comparison of risk assessment based on the various methods of heavy metals in soil: A case study for the typical field areas in the Jianghan Plain. Hydrogeol. Eng. Geol..

[16] Geng Y., Zhang C., Zhang Y., Huang D., Yan Z., Sun T., Cheng L., Wang J., Mao Y. (2021). Fugitive patterns and ecological risk assessment of heavy metals in urban sludge in China. Chin. J. Environ. Sci..

[17] Matong J., Nyaba L., Nomngongo P.N. (2016). Fractionation of trace elements in agricultural soils using ultrasound assisted se-quential extraction prior to inductively coupled plasma mass spectrometric determination. Chemosphere.

[18] Saeedi M., Jamshidi Zanjani A. (2015). Development of a new aggregative index to assess potential effect of metals pollution in aquatic sediments. Ecol. Indic..

[19] Soto Jiménez M.F., Olvera Balderas D. (2018). Geochemical Fractionation and Potential Ecological Risk of Cadmium and Lead in Soils Impacted by Secondary Lead Refinery. Bull. Environ. Contam. Toxicol..

[20] Zhu H., Yuan X., Zeng G., Jiang M., Liang J., Zhang C., Yin J., Huang H., Liu Z., Jiang H. (2012). Ecological risk assessment of heavy metals in sediments of Xiawan Port based on modified potential ecological risk index. Trans. Nonferrous Met. Soc. China.

[21] Xiong H., Chen J. (2020). A study on ecological risk assessment of soil heavy metals based on Monte Carlo-Hakanson simulation. J. Agro-Environ. Sci..

[22] Pang K., Li M., Liu L., Yang J., Zhao H. (2022). Heavy metal pollution assessment and source analysis of sediments in the Yellow River basin based on Monte Carlo simulation and PMF model. Chin. J. Environ. Sci..

[23] Li J., Dong H., Xu X., Han B., Li X., Zhu C., Han C., Liu S., Yang D., Xu Q. (2016). Prediction of the bioaccumulation of PAHs in surface sediments of Bohai sea, China and quantitative assessment of the related toxicity and health risk to humans. Mar. Pollut. Bull..

[24] Jia S., Sankaran G., Wang B., Shang H., Tan S.T., Yap H.M., Shen J., Gutiérrez R.A., Fang W., Liu M. (2019). Exposure and risk assessment of volatile organic compounds and airborne phthalates in Singapore′s Child care centers. Chemosphere.

[25] Noutsopoulos C., Koumaki E., Vasileios S., Mamais D. (2019). Analytical and mathematical assessment of emerging pollutants fate in a river system. J. Hazard. Mater..

[26] Zhang H., Zhou X., Wang L., Wang W., Xu J. (2018). Concentrations and potential health risks of strontium in drinking water from Xi’an, Northwest China. Ecotoxicol. Environ. Saf..

[27] Chen R., Han L., Liu Z., Zhao Y., Li R., Xia L., Fan Y. (2022). Assessment of Soil-Heavy Metal Pollution and the Health Risks in a Mining Area from Southern Shaanxi Province, China. Toxics.

[28] (2008). Soil Quality-Analysis of Total Mercury, Arsenic and Lead Contents-Atomic Fluorescence Spectrometry-Part2: Analysis of Total Arsenic Contents in Soils.

[29] (2015). Solid Waste–Determination of Metals–Inductively Coupled Plasma Mass Spectrometry.

[30] Xing H.L. (2016). Analysis of Soil Element Endowment Patterns and Ecological Risk Assessment in Anxin-Qingyuan County, Hebei.

[31] (2010). Extraction Procedure for the Morphological Order of 13 Trace Elements in Soils and Sediments.

[32] Demirak A., Kocakaya M., Keskin F. (2022). Chemical fractions of toxic metals and assessment of risks on the environment and health in Mugla topsoils. Int. J. Environ. Sci. Technol..

[33] Rauret G., Lopez-Sanchez J.F., Sahuquillo A. (1999). Improvement of the BCR three step sequential extraction procedure prior to the certification of new sediment and soil reference materials. J. Environ. Monit..

[34] (2003). Certificate of Standard Substances Standard Substances for Soil Composition Analysis.

[35] Müller G. (1986). Index of geoaccumulation in sediments of the Rhine River. Geol. J..

[36] Loska K., Wiechula D., Barska B., Cebula E., Chojnecka A. (2003). Assessment of arsenic enrichment of cultivated soils in southern Poland. Pol. J. Environ. Stud..

[37] Li Z., Ma Z., van der Kuijp T.J., Yuan Z., Huang L. (2014). A review of soil heavy metal pollution from mines in China: Pollution and health risk assessment. Sci. Total Environ..

[38] Xue C., Xiao L., Wu Q., Li D., Wang K., Li H., Wang R. (1986). A study on the background values of ten elements in major agri-cultural soils in Shaanxi Province. J. Northwest Sci.-Tech. Univ. Agric. For. (Nat. Sci. Ed.).

[39] Förstner U., Ahlf W., Calmano W., Kersten M. (1990). Sediment criteria development. Sediments and Environmental Geochemistry.

[40] Si M., Wang Z., Wang J. (2019). Progress in the application of Hakanson index method in assessing ecological risk of soil heavy metals. Chin. J. Soil Sci..

[41] Xiang Q., Yu H., Chu H., Hu M., Xu T., Xu X., He Z. (2022). The potential ecological risk assessment of soil heavy metals using self-organizing map. Sci. Total Environ..

[42] Li X., Xu C., Liu X., Liu J., Zhang X. (2015). Biological activity and environmental risk of heavy metals in urban soils of Baoji. Acta Sci. Circumst..

[43] Lu C., Li T., Fu Y., Xu Y., Zhang J. (2015). Creation of Hakanson’s potential ecological risk index method based on bioavailability and wide concentration range: An example of farmland soils in the Xiaoqinling gold mining area. Geol. Bull. China..

[44] Xiao R., Guo D., Ali A., Mi S., Liu T., Ren C., Li R., Zhang Z. (2019). Accumulation, ecological-health risks assessment, and source apportionment of heavy metals in paddy soils: A case study in Hanzhong, Shaanxi, China. Environ. Pollut..

[45] Hakanson L. (1980). An ecological risk index for aquatic pollution control: A sedimentological approach. Water Res..

[46] Ma J., Han C., Jiang Y. (2020). Some issues in the application of potential ecological risk index method. Geogr. Res..

[47] Li Y., Ma J., Liu D. (2015). Assessment of heavy metal pollution and potential ecological risk in Kaifeng urban soils. Chin. J. Environ. Sci..

[48] U.S. Environmental Protection Agency (2001). Risk Assessment Guidance for Superfund Volume III: Part A, Process for Conducting Probabilistic Risk Assessment.

[49] Xiong H., Zhang H., Chen S. (2022). A study on the health risk assessment of heavy metals in soil of a contaminated site based on Monte Carlo simulation. J. Hefei Univ. Technol. (Nat. Sci. Ed.).

[50] China National Environmental Monitoring Centre (1990). Background Values of Soil Elements in China.

[51] Wang R., Chen M., Chen N., Liu G., Zhang E., Liu X., Zhang J. (2017). Comparison of ecological risk assessment of soil heavy metals based on total amount and morphology: A case study of Sizhong Township, Longyan City. Chin. J. Environ. Sci..

[52] Tong S.M., Yang L.S., Gong H.Q., Wang L., Li H.R., Yu J.P., Li Y.H., Deji Y.Z., Nima C.J., Zhao S.C. (2022). Bioaccumulation characteristics, transfer model of heavy metals in soil-crop system and health assessment in plateau region, China. Ecotoxicol. Environ. Saf..

[53] Ji Z., Long Z., Zhang Y., Wang Y., Qi X., Xia X., Pei Y. (2021). Enrichment differences and source apportionment of nutrients, stable isotopes, and trace metal elements in sediments of complex and fragmented wetland systems. Environ. Pollut..

[54] Jiang Y., Zhou H., Gu J.F., Zeng P., Liao B.H., Xie Y.H., Ji X.H. (2022). Combined amendment improves soil health and brown rice quality in paddy soils moderately and highly co-contaminated with Cd and As. Environ. Pollut..

[55] Ma X., Zuo H., Tian M., Zhang L., Meng J., Zhou X., Min N., Chang X., Liu Y. (2016). Assessment of heavy metals contamination in sediments from three adjacent regions of the Yellow River using metal chemical fractions and multivariate analysis techniques. Chemosphere.

[56] Chen L., Liu J.R., Hu W.F., Gao J., Yang J.Y. (2021). Vanadium in soil-plant system: Source, fate, toxicity, and bioremediation. J. Hazard. Mater..

[57] Nemati K., Bakar N.K.A., Sobhanzadeh E., Abas M.R. (2009). A modification of the BCR sequential extraction procedure to inves-tigate the potential mobility of copper and zinc in shrimp aquaculture sludge. Microchem. J..

[58] Iwegbue C.M.A., Eghwrudje M.O., Nwajei G.E., Egboh S.H.O. (2007). Chemical speciation of heavy metals in the Ase River sed-iment, Niger Delta, Nigeria. Chem. Spec. Bioavailab..

[59] Liu B., Luo J., Jiang S., Wang Y., Li Y., Zhang X., Zhou S. (2021). Geochemical fractionation, bioavailability, and potential risk of heavy metals in sediments of the largest influent river into Chaohu Lake, China. Environ. Pollut..

[60] Moore F., Nematollahi M.J., Keshavarzi B. (2014). Heavy metals fractionation in surface sediments of Gowatr bay-Iran. Environ. Monit. Assess..

[61] Jiang L., Sun H., Peng T., Ding W., Liu B., Liu Q. (2021). Comprehensive evaluation of environmental availability, pollution level and leaching heavy metals behavior in non-ferrous metal tailings. J. Environ. Manag..

[62] Li S.M., Wu Y.Y., Wu Z.P., Wang Q.C., Li C.S., Hou Z.W., Fu Z.L. (2023). Effects of different organic amendments on dissolved organic matter and lead occurrence formation in soil of mining areas. J. Agric. Resour. Environ..

[63] Lanno R.P., Oorts K., Smolders E., Albanese K., Chowdhury J. (2019). Effects of soil properties on the toxicity and bioaccumulation of lead in soil invertebrates. Environ. Toxicol. Chem..

[64] Li C., Sanchez G.M., Wu Z., Cheng J., Zhang S., Wang Q., Li F., Sun G., Meentemeyer R.K. (2020). Spatiotemporal patterns and drivers of soil contamination with heavy metals during an intensive urbanization period (1989–2018) in southern China. Environ. Pollut..

[65] Wojtkowska M., Bogacki J., Witeska A. (2016). Assessment of the hazard posed by metal forms in water and sediments. Sci. Total Environ..

[66] Elouear Z., Bouhamed F., Boujelben N., Bouzid J. (2016). Assessment of toxic metals dispersed from improperly disposed tailing, Jebel Ressas mine, NE Tunisia. Environ. Earth Sci..

[67] Khoeurn K., Sakaguchi A., Tomiyama S., Igarashi T. (2019). Long-term acid generation and heavy metal leaching from the tailings of Shimokawa mine, Hokkaido, Japan: Column study under natural condition. J. Geochem. Explor..

[68] Dubey S., Shri M., Gupta A., Rani V., Chakrabarty D. (2018). Toxicity and detoxification of heavy metals during plant growth and metabolism. Environ. Chem. Lett..

[69] Sonter L.J., Ali S.A., Watson J.E.M. (2018). Mining and biodiversity: Key issues and research needs in conservation science. Proc. Biol. Sci..

[70] Barcelos D.A., Pontes F.V.M., da Silva F.A.N.G., Castro D.C., dos Anjos N.O.A., Castilhos Z.C. (2020). Gold mining tailing: En-vironmental availability of metals and human health risk assessment. J. Hazard. Mater..

