# Supplementary material for: Probabilistic Risk Assessment of Heavy Metals in Mining Soils Based on Fractions: A Case Study in Southern Shaanxi, China
Source: Toxics. 2023 Dec 7;11(12):997. doi: 10.3390/toxics11120997 (PMC10748273; doi:10.3390/toxics11120997)
Supplement: Supplementary file 1 [file toxics-11-00997-s001.zip › toxics-2733619-supplementary.pdf]

Supplementary Materials

Probabilistic risk assessment of heavy metals in mining soils based on fractions: A case study in southern Shaanxi, China

Lei Han[1,2*], Yamin Fan[1], Rui Chen[3], Yunmeng Zhai[1], Zhao Liu[1,2], Yonghua Zhao[1,2], Risheng Li[4], Longfei Xia[4]

1 School of Land Engineering, Chang'an University, Xi'an 710054, China

2 Shaanxi Key Laboratory of Land Consolidation, Xi'an 710054, China

3 School of Earth Science and Resources, Chang'an University, Xi'an 710054, China

4 Shaanxi Provincial Land Engineering Construction Group, Xi'an 710075, China

***Corresponding author:** School of Land Engineering, Chang'an University, Xi'an 710054, China

E-mail addresses: hanshuanglei@chd.edu.cn (L. H.)

Tables: 7

Figures: 2

Pages: 8

Tables

Table S1. Modified BCR sequential extraction procedure

Table S2. The modified Hakanson index method risk degree classification standard

Table S3. Environmental background values for heavy metals (HMs) in yellow-brown loam

Table S4. Concentrations of total heavy metals in the study area

Table S5. Concentrations of the heavy metal fractions in the study area

Table S6. Comparison of potential ecological risk of HMs in soil based on the Hakanson index and modified Hakanson index

Table S7. Probability of the ecological hazard index of HMs at different risk degrees (%)

Figures

Figure S1. Cumulative frequencies distribution of ecological risk index of heavy metals

Figure S2. Sensitivity analysis of comprehensive ecological hazard index

Table S1. Modified BCR sequential extraction procedure [1-3]

Fractions	Extractant	Equilibrium condition
F1	40ml 0.11mol/L Hac boiling cooling, pH 7.0 water	Shake 16h, centrifuge 20min, supernatant to be tested
F2	40ml 0.5mol/L $NH_2OH{\cdot}HCl$	
F3	10ml H_2O_2/10ml H_2O_2 50ml 1mol/L NH_4Ac	Digestion at 25 °C and water bath for 1 h each； Shake 16h, centrifuge 20min, supernatant to be tested
F4	HCl-HNO_3-HF	0.1g dried solid residue microwave digestion constant volume standby

Table S2. The modified Hakanson index method risk degree classification standard [4,5]

Risk Degree	Low	Moderate	Considerate	High	Very high
E_r^i	<10	10-20	20-40	40-80	>80
**RI**	<30	30-60	60-120	120-240	>240

Table S3. Environmental background values for heavy metals (HMs) in yellow-brown loam [6]

HMs	Min/ mg/kg	Max/ mg/kg	Mean/ mg/kg	SD
Cr	8.0	275.6	66.9	25.75
Ni	1.0	142	31.5	14.73
Cu	5.0	144.6	23.4	10.84
Zn	22.9	283.0	71.8	24.07
As	0.7	89.5	11.8	6.21
Pb	11.1	234.0	29.2	12.1

Table S4. Concentrations of total heavy metals in the study area

HMs	Max/ mg/kg	Min/ mg/kg	Mean/ mg/kg	SD	CV/%
Cr	136.76	42.55	89.62	32.63	36
Ni	64.08	38.08	47.57	6.80	14
Cu	83.91	36.67	54.00	13.82	26
Zn	141.76	70.86	110.08	22.29	20
As	2030.70	51.11	1080.83	695.05	64
Pb	72.90	30.86	45.73	12.03	26

Table S5. Concentrations of the heavy metal fractions in the study area

HMs	Fractions	Max/ mg/kg	Min/ mg/kg	Mean/ mg/kg	SD	CV/%	S-W Test	Significance
Cr	F1	62.66	2.10	26.80	17.92	66.88	Normal	0.391
	F2	2.39	0.01	1.40	0.59	42.35	Normal	0.542
	F3	2.80	0.01	0.60	0.86	142.00	Lognormal	0.000
	F4	87.68	30.87	60.82	19.80	32.55	Normal	0.126
Ni	F1	31.07	1.76	8.52	6.76	79.42	Lognormal	0.000
	F2	8.40	3.16	5.65	1.65	29.13	Normal	0.506
	F3	5.00	1.47	3.17	1.16	36.76	Normal	0.286
	F4	38.70	17.05	30.05	5.95	19.81	Normal	0.437

Cu	F1	31.16	3.56	12.92	7.83	60.62	Lognormal	0.027
	F2	44.59	3.10	11.76	9.39	79.81	Lognormal	0.000
	F3	4.82	0.10	1.08	1.45	133.44	Lognormal	0.000
	F4	47.83	14.16	28.23	8.70	30.82	Lognormal	0.036
Zn	F1	66.65	19.19	37.47	12.56	33.52	Normal	0.188
	F2	17.59	3.59	9.09	3.94	43.34	Normal	0.479
	F3	5.38	0.41	1.93	1.28	66.19	Normal	0.077
	F4	93.54	33.73	62.40	17.26	27.65	Normal	0.610
As	F1	886.71	23.90	346.56	283.55	81.82	Normal	0.132
	F2	1024.77	0.38	266.41	291.73	109.51	Abnormal	0.006
	F3	7.68	0.00	2.96	2.52	85.32	Normal	0.101
	F4	1062.57	8.48	464.90	506.27	108.90	Lognormal	0.004
Pb	F1	15.39	0.27	5.03	4.37	86.87	Lognormal	0.039
	F2	31.57	1.04	12.99	7.17	55.19	Normal	0.136
	F3	0.43	0.01	0.11	0.13	115.40	Lognormal	0.002
	F4	64.79	13.95	27.59	13.88	50.31	Lognormal	0.004

Table S6. Comparison of potential ecological risk of HMs in soil based on the Hakanson index and modified Hakanson index

	**The ecological hazard index**	**Cr**	**Ni**	**Cu**	**Zn**	**As**	**Pb**
Hakanson index method	E_r^i (Max)	4.08	9.45	15.87	1.96	1440.21	21.45
	E_r^i (Min)	1.27	5.62	6.94	0.98	36.25	9.08
	E_r^i (Mean)	2.68	7.02	10.21	1.52	766.54	13.45
	Risk degree	Low	Low	Moderate	Low	Very high	Moderate
	RI (Mean)	801.43					
	Risk degree	Very high					
Modified	E_r^i (Max)	4.23	10.70	15.59	2.15	1602.96	15.36

Hakanson index method	E_r^i (Min)	2.23	5.06	6.25	1.03	49.87	7.36
	E_r^i (Mean)	2.96	6.08	9.54	1.50	765.84	11.09
	Risk degree	Low	Low	Low	Low	Very high	Moderate
	RI (Mean)	797.00					
	Risk degree	Very high					

Table S7. Probability of the ecological hazard index of HMs at different risk degrees (%).

**Risk Degree**	**Cr**	**Ni**	**Cu**	**Zn**	**As**	**Pb**	**RI**
Low	100.00	97.21	64.75	100.00	7.51	36.15	7.28
Moderate	0	2.63	34.89	0	0.21	63.85	0.65
Considerate	0	0.15	0.37	0	0.44	0	1.41
High	0	0.01	0	0	0.93	0	3.99
Very high	0	0	0	0	90.91	0	86.66

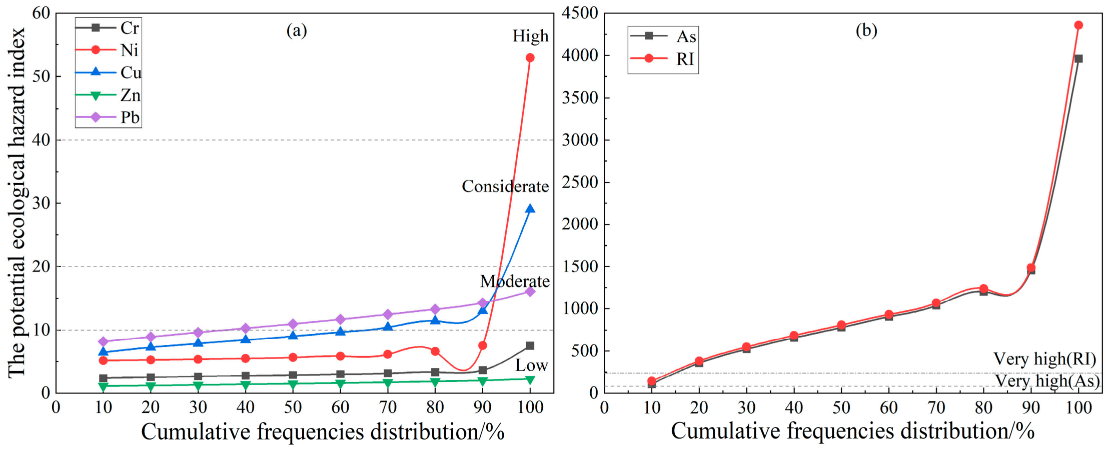

Figure S1. Cumulative frequencies distribution of ecological risk index of (a) Cr, Ni, Cu, Zn and Pb; (b) As and RI

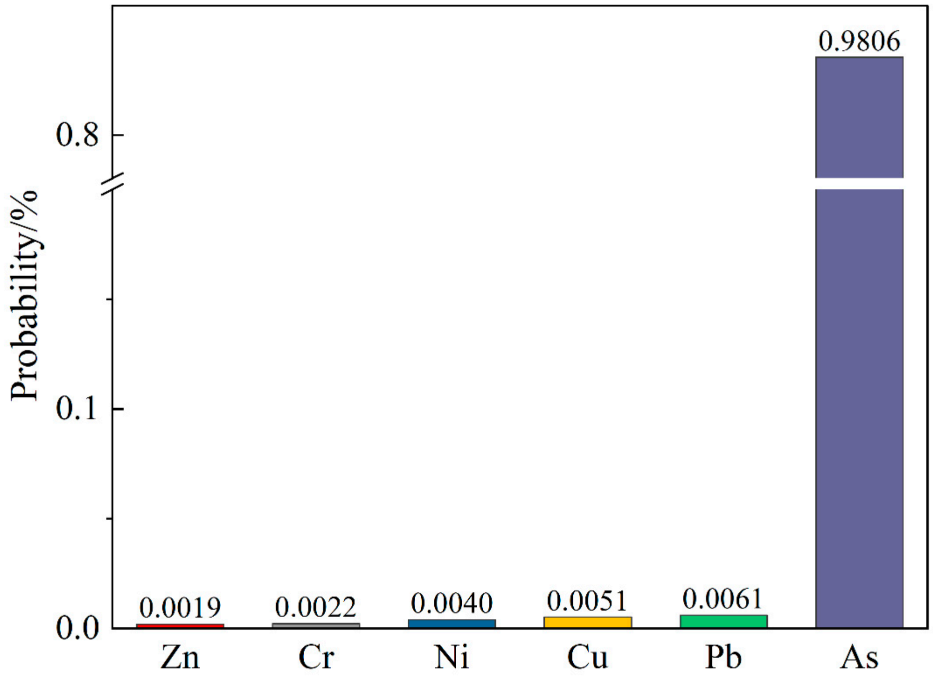

Figure S2. Sensitivity analysis of comprehensive ecological hazard index

References

1. GB/T 25282-2010: Extraction procedure for the morphological order of 13 trace elements in soils and sediments. Ministry of Land and Resources of the People's Republic of China, **2010**. https://www.instrument.com.cn/download/shtml/186602.shtml. Accessed on 10 November 2010.
2. Demirak, A.; Kocakaya, M.; Keskin, F. Chemical fractions of toxic metals and assessment of risks on the environment and health in Mugla topsoils. *Int J Environ Sci Technol*, **2022**, *19 (6)*: 5631–5648.
3. Rauret, G.; Lopez-Sanchez, J.F.; Sahuquillo, A. Improvement of the BCR three step sequential extraction procedure prior to the certification of new sediment and soil reference materials. *J Environ Monit* **1999**, *1(1)*: 57-61
4. Ma, J.; Han, C.; Jiang, Y. Some issues in the application of potential ecological risk index method. *Geogr Res* **2020**, *39(06)*: 1233-1241.
5. Li, Y.; Ma, J.; Liu, D. Assessment of heavy metal pollution and potential ecological risk in Kaifeng urban soils. *Chin J Envir Sci* **2015**, *36(03)*: 1037-1044
6. China National Environental Monitoring Centre. Background values of soil elements in China: Beijing, China, **1990**.
